# Routine evaluation of tonsillectomy specimens: a cross-sectional survey of Canadian Otolaryngology: Head and Neck Surgeons

**DOI:** 10.1186/s40463-022-00569-7

**Published:** 2022-04-06

**Authors:** Alexi Kuhnow, Ahmed A. Al-Sayed, Benjamin Taylor

**Affiliations:** 1 Faculty of Medicine, Dalhousie University, 1459 Oxford Street, Halifax, NS B3H 4R2 Canada; 2 Division of Otolaryngology – Head and Neck Surgery, Nova Scotia Health Authority, 5820 University Ave, Halifax, NS Canada

**Keywords:** Routine tonsillectomy, Tonsillectomy specimen, Occult malignancy, Survey, Clinical practice, Otolaryngology

## Abstract

**Background:**

Tonsillectomy is a commonly performed procedure in Canada. The rate of occult malignancy is rare in adult and pediatric populations. At present, no guidelines exist surrounding the need for routine histopathological evaluation of tonsil specimens when no malignancy is suspected.

**Methods:**

We sent a confidential online survey to active members of the Canadian Society of Otolaryngology – Head and Neck Surgery (CSO-HNS) about their current tonsillectomy practice and beliefs surrounding the need for routine histopathological evaluation of tonsillectomy specimens when no malignancy is suspected. We used Opinio survey software for data collection and descriptive statistics.

**Results:**

95 participants completed our survey (response rate 19.3%). Most participants reported performing both adult and pediatric tonsillectomies. When no malignancy is suspected, participant responses were split between whether they send tonsil specimens in pediatrics only (4.2%), in adults only (31.6%), or not sending specimens (29.5%). Half of the participants reported that routinely sending specimens to rule out occult malignancy is an institutional policy. Approximately 75% of participants were in favour of removing this practice in both the pediatric and adult populations.

**Conclusion:**

Eliminating the practice of automatically sending tonsil specimens for histopathological evaluation when no malignancy is suspected was supported by the majority of study participants. This is in keeping with Choosing Wisely, a campaign designed to facilitate conversations about unnecessary medical tests and procedures. Institutional change is likely required in order to alter this practice.

**Graphical Abstract:**

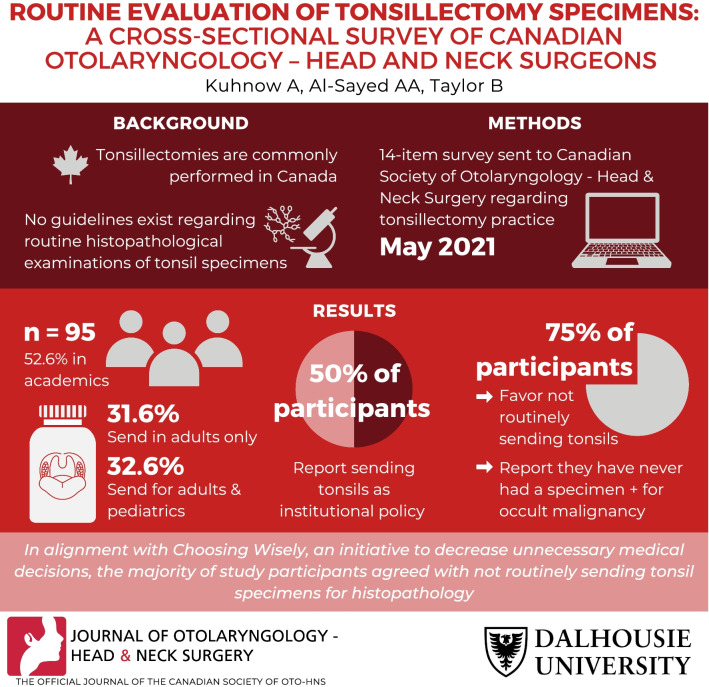

**Supplementary Information:**

The online version contains supplementary material available at 10.1186/s40463-022-00569-7.

## Background

Tonsillectomy is a commonly performed surgical procedure. In Ontario, 14,000 pediatric tonsillectomies are performed each year [1]. The two most common indications for tonsillectomy in both children and adults are obstructive sleep apnea and recurrent or chronic infection [2–4]. Tonsillectomy is less commonly performed for suspicion of malignancy [5].

Historically, it was suggested that every specimen should undergo histopathological (HP) evaluation in order to identify occult malignancy [6, 7]. However, automatically sending all surgical specimens for analysis is a resource-intensive practice. Many centers no longer suggest routine analysis of specimens unless the results of analysis are likely to change patient care [3, 6, 7].

The rate of occult malignancy found in both adult and pediatric tonsillectomy specimens is very low. Rates of clinically occult tonsil malignancy in all age groups ranged from 0.01% in a meta-analysis [8] to 0.015% in a systematic review [9]. An American study of 1746 adult tonsillectomy specimens found no occult malignancy [3]. Despite increasing research in this area, there are no clinical practice guidelines concerning the need for routine histopathological analysis of tonsillectomy specimens when no malignancy is suspected. Furthermore, in Canada little is known about whether these specimens are routinely sent for HP evaluation.

Our main objectives were to (1) determine the clinical practices of Canadian Otolaryngology – Head & Neck (OTO-HN) surgeons requesting histopathological analysis of tonsillectomy specimens when no malignancy is suspected, and (2) assess whether a consensus could be reached to discontinue this practice. This could inform clinical practice guidelines, and is in keeping with Choosing Wisely’s mandate to reduce unnecessary testing in healthcare [10, 11].

## Methods

### Participants

Active members of the Canadian Society of Otolaryngology – Head and Neck Surgery (CSO-HNS) were invited to participate. Our inclusion criteria were that participants had to be currently practicing OTO-HN surgery as an attending physician. Associate membership in the CSO-HNS is exclusively for resident physicians, and affiliate membership is for individuals working in related fields who may not be physicians; therefore, these members were excluded. There were no other exclusion criteria because our sample population consisted of physicians who met our inclusion criteria.

### Survey

We developed our survey using the guide by Burns et al. for self-administered surveys of clinicians [12]. We generated items through literature review. Four domains emerged: demographics, current practices in adult tonsillectomies, current practices in pediatric tonsillectomies, and beliefs and attitudes towards routine evaluation of tonsillectomy specimens. We reduced items to eliminate redundancies but maintain questions in these domains. We designed our survey to be quick to complete in order to encourage participation.

Our final confidential 14-item survey was populated into the survey software Opinio (ObjectPlanet Inc., Oslo, Norway) in order to be provided online. Opinio also provided descriptive statistics. In order to ask participants about their current clinical practices, we asked how many tonsillectomies participants perform as an open-ended question. Next, participants could select whether they perform adult tonsillectomies only, pediatric tonsillectomies only, both pediatric and adult tonsillectomies, or that they do not perform tonsillectomies. We asked participants whether they send tonsil specimens for evaluation when no malignancy is suspected, and multiple-choice options were given. These included “yes, for pediatric tonsillectomies only”, “yes, for adult tonsillectomies only”, “yes, for both adult and pediatric tonsillectomies”, “no” and “not applicable”. We also asked a yes or no question of whether this practice was an institutional policy. We then asked participants how many specimens to their recollection had returned with occult malignancy in their years of practice, which was open-ended. We did not ask them to specify whether any risk factors for malignancy were present.

We asked participants about their beliefs and attitudes about sending tonsil specimens when no malignancy is suspected. Participants could rate their agreement from “strongly disagree” to “strongly agree” with the following two statements: “In pediatrics, tonsil specimens should be evaluated when no malignancy is suspected” and “In adults, tonsil specimens should be evaluated when no malignancy is suspected”. Lastly, participants were invited to write any additional comments they had. Please see Additional file 1: Appendix I - Survey Questions for the questions that we asked participants.

We performed an a priori power calculation to assess how many survey responses were needed in order to provide sufficient statistical accuracy. Using 95% confidence intervals and a 10% margin of error, we determined that a response rate of approximately 18% was required.

Invitations to participate were sent by email in May 2021 by the CSO-HNS to 493 active members. One reminder email was sent in June in order to increase the response rate. Participants provided written informed consent before being able to access the online survey. Our study was approved by the Nova Scotia Health Authority Research Ethics Board (File #1026512).

## Results

### Survey response

Of the 493 OTO-HN surgeons who were invited to participate, 95 completed the survey (response rate = 19.3%). This met our criteria for a sufficiently powered survey.

### Demographics

Table 1—Demographics of Participants has full demographics information. Participants were from across Canada. Approximately half (52.6%) practice in an academic centre. Most participants were male (68.4%) and either 30–39 years old (30.5%) or 40–49 years old (33.7%). The majority of participants have practiced OTO-HNS for either 10–20 years (31.6%), or over 20 years (30.5%).

**Table 1:** Demographics

	Number (%)
**Main practice area**	
Alberta	13 (13.7)
British Columbia	12 (12.6)
Manitoba	5 (5.3)
New Brunswick	4 (4.2)
Nova Scotia	9 (9.5)
Ontario	31 (32.6)
Prince Edward Island	1 (1)
Quebec	14 (14.7)
Saskatchewan	3 (3.2)
Other	3 (3.2)
**Practice setting**	
Academic	50 (52.6)
Not Academic	45 (47.4)
**Age range**	
< 30	0
30–39	29 (30.5)
40–49	32 (33.7)
50–59	15 (15.8)
60–69	18 (19)
> 70	1 (1)
**Gender**	
Male	65 (68.4)
Female	29 (30.5)
Other	0
Prefer not to answer	1 (1)
**Fellowship training**	
Yes	59 (62.1)
No	32 (33.4)
Not specified	4 (4.2)
**Years in practice**	
< 5 years	23 (24.2)
5–9 years	13 (13.7)
10–20 years	30 (31.6)
> 20 years	29 (30.5)

### Current practices in tonsillectomy

Table 2—Tonsillectomy Practices reports details we asked about tonsillectomy practices. More than 30% of participants report removing at least 100 tonsils per year (31.6%), 28.4% report removing 50–99 per year, 24.2% remove 10–49 per year and 15.8% of participants remove less than 10 per year. More than two thirds of participants perform both pediatric and adult tonsillectomies (65.3%).

**Table 2:** Tonsillectomy Practices

	Number (%)
*Yearly tonsillectomies performed	
< 10	15 (15.8)
10–49	23 (24.2)
50–99	27 (28.4)
> 100	30 (31.6)
**Perform**	
Adult tonsillectomies only	22 (23.2)
Pediatric tonsillectomies only	8 (8.4)
Both pediatric and adult tonsillectomies	62 (65.3)
No tonsillectomies	3 (3.2)
**Send specimens for evaluation when NO malignancy suspected**	
Yes, for pediatric tonsillectomies only	4 (4.2)
Yes, for adult tonsillectomies only	30 (31.6)
Yes, for both adult and pediatric tonsillectomies	31 (32.6)
No	28 (29.5)
Not applicable	2 (2.1)
**Is sending routine tonsil specimens an institutional policy?**	
Yes	48 (50.5)
No	47 (49.5)
*If specimens sent, how many have had occult malignancy?	
0	48 (73.9)
1	5 (7.7)
2–5	3 (4.6)
6–10	0
> 10	2 (3.1)
Not answered	5 (7.7)

*Asked as an open-ended question

With respect to sending tonsil specimens for evaluation when no malignancy is suspected results were varied. A third of participants send specimens in adults only (31.6%), 32.6% send them for both adults and pediatrics, and 29.5% do not routinely send specimens when no malignancy is suspected. Four participants (4.2%) report sending specimens only for pediatric tonsillectomies. This practice is reported as an institutional policy for half of participants (50.5%).

We asked participants how many times a routine tonsillectomy specimen that had been sent for HP evaluation returned with occult malignancy over their years of practice. The majority of participants answered none (73.9%); however, 7.7% recalled one specimen returning positive, and 4.6% recalled 2–5. One participant reported 12 specimens had returned with occult malignancy, and another reported 30. Both of these participants reported performing over 100 tonsillectomies per year.

### Beliefs and attitudes about routine tonsil specimen evaluation

We asked participants about their agreement with the statement, “In pediatrics, tonsil specimens should be evaluated when no malignancy is suspected.” Forty participants strongly disagreed with this statement. Thirty-two disagreed, 10 were neutral, 8 agreed and 5 strongly agreed. We also asked the same question in adults, and the number of participants selecting each response was the same. Please see Table 3—Beliefs and Attitudes about Sending Tonsillectomy Specimens for Routine Evaluation for the percentage of participants who selected each answer.

**Table 3:** Beliefs and Attitudes

	Number (%)
**In pediatrics, tonsil specimens should be evaluated when NO malignancy suspected**	
Strongly disagree	40 (42.1)
Disagree	32 (33.7)
Neither agree nor disagree	10 (10.5)
Agree	8 (8.4)
Strongly agree	5 (5.3)
**In adults, tonsil specimens should be evaluated when NO malignancy suspected**	
Strongly disagree	40 (42.1)
Disagree	32 (33.7)
Neither agree nor disagree	10 (10.5)
Agree	8 (8.4)
Strongly agree	5 (5.3)

We also asked an open-ended question about participants’ thoughts on routinely sending tonsil specimens for evaluation when no malignancy is suspected. Out of the total of 95 participants, 23 left comments. Multiple themes emerged from their responses, and participant statements were diverse. Table 4—Themes about Sending Tonsil Specimens for Evaluation provides further details on the themes that emerged from these comments. In terms of current practices, 7 participants expressed that sending specimens was an institutional policy. One participant reported that this was provincial policy, another reported it was based on the ability to bill for tonsillectomy, and two reported that their practices were based on previous experience or the experience of a colleague.

**Table 4:** Themes

Themes/comments	Number of times theme mentioned
**Current practices**	
Practice based on institutional policy	7
Practice based on provincial policy	1
Practice based on ability to bill for tonsillectomy	1
Practice based on analogy/previous experience	2
**Possible practice changes**	
Willing to change practice based on consensus	1
Should send specimen if patient > 30	4
Should send specimen if patient > 40	2
Should send specimens due to rising HPV prevalence	3
No role for routine evaluation	1
**Concerns**	
Medicolegal impact of not sending	1
**Beliefs**	
Every tissue removed from body requires evaluation	1
Total number of survey responses	95
Total number of participants who left comments	23

*HPV* human papilloma virus

Ideas also emerged surrounding what the policy should be. One participant wrote they were willing to change their practice based on the evidence and consensus. Four participants wrote that tonsil specimens should be sent for patients over 30 years old, and 2 participants wrote that they should be sent if patients were over the age of 40. Three responses indicated that tonsil specimens should be sent due to the rising prevalence of human papilloma virus. Finally, one participant expressed that there is no role for routine evaluation based on the current evidence.

## Discussion

To our knowledge, this is the first study to survey Canadian OTO-HN surgeons about their practices surrounding routine HP evaluation of tonsillectomy specimens when no malignancy is suspected. Overall, the response rate was 19.3%. Response rates for online surveys of surgeons vary widely, with reports from 9 to 80% [13].

Multiple studies have recommended against routinely sending tonsillectomy specimens for evaluation when no malignancy is suspected based on the very low rates of occult malignancy [3, 8, 9, 14]. However, to our knowledge there are no consensus guidelines that specify what should be done with routine tonsillectomy specimens in Canada.

Current evidence supports discontinuing routine HP analysis of tonsil specimens after tonsillectomy when no malignancy is suspected. Criteria have been established for what risk factors may lead a surgeon to suspect malignancy prior to tonsillectomy. As shown in Table 5—Risk Factors for Occult Malignancy in Tonsillectomy Specimens, these include: history of head and neck cancer/radiation, immunodeficiency, visible tonsillar asymmetry or abnormal appearance, cervical lymphadenopathy, unexplained weight loss, and constitutional symptoms [9, 14, 15]. When malignancy is not suspected, meaning the above risk factors are not present, the rate of occult malignancy is rare at less than 0.015% for all age groups [8, 9], and 0% for adults across multiple studies [3, 14].

**Table 5:** Risk Factors

Risk factors
1. History of head and neck cancer
2. Visible tonsillar asymmetry or abnormality
3. Cervical lymphadenopathy
4. Unexplained weight loss
5. Constitutional symptoms

These rates are fairly congruent with the number of occult malignancies reported by participants in our survey. Approximately 75% of participants reported that in their years of practice, no specimens had returned positive for occult malignancy. Ten participants reported that they had had specimens return with occult malignancy, but we did not specify in our survey whether they recalled if risk factors as outlined above were present in these cases. There were two participants who reported 12 and 30 specimens returning with occult malignancy. This could be because of a high volume of tonsillectomies performed (they each reported > 100 per year), or it could be due to other factors that we did not assess, such as having an oncology-focused practice. Further, we did not ask whether these patients had any of the risk factors in Table 5 that may lead a surgeon to suspect malignancy.

Over 75% of participants selected that they disagree/strongly disagree that tonsil specimens should be evaluated with no malignancy is suspected, both in adult and pediatric patients. However, about 60% of participants reported sending routine tonsillectomy specimens for analysis. Half of the total participants reported that this practice was an institutional policy, which may partially account for the discrepancy between participant beliefs and current practices. From the open-ended question in our survey, other reasons for continuing this practice could be that it’s part of provincial policy, and that billing for tonsillectomy requires sending specimens. These reasons all point to governmental or institutional policies playing a role in continuing a practice that is no longer supported by evidence.

There were 13 (13.6%) participants who selected agree/strongly agree when asked if tonsil specimens should be evaluated when no malignancy is suspected in both adults and pediatrics. Multiple reasons for this emerged from the 23 responses that were left in the comments section of our survey. First, patient age was a deciding factor to send specimens. Some participants advocated for routinely sending specimens for patients over 30 or 40. Other responses identified that the increasing prevalence of human papilloma virus is a reason to continue sending specimens for evaluation of occult malignancy.

Sending routine tonsillectomy specimens for HP analysis seems to be functioning as a screening test for malignancy, since no malignancy is suspected in these cases. When viewed in this light, this practice is a poor screening test. First, it’s expensive, especially given the large number of routine tonsillectomies performed yearly. A Canadian study in 2015 calculated the cost of HP analysis as $128.65 for bilateral tonsil specimens [14]. Eliminating the practice would be in keeping with the Choosing Wisely mandate to reduce unnecessary spending in healthcare [10]. Further, sending tonsil specimens has a poor number needed to screen to detect one case of occult malignancy. The same 2015 study estimated that the number needed to screen was 3904. A more reasonable approach is likely to assess patients undergoing tonsillectomy for the five risk factors in Table 5. This would increase the rate of malignancy detected, and in this scenario HP analysis would no longer be a screening test.

Given that most participants were in favour of discontinuing HP analysis for routine tonsillectomies, this practice could be added to the list on the Choosing Wisely Canada website titled “Three things physicians and patients should question in Otolaryngology—Head and Neck Surgery” [16]. This could help to inform institutional policy, and lay the groundwork for eliminating a practice that is not supported in the current body of literature.

### Limitations

There were multiple limitations in our study. The first is that surveys have naturally occurring biases including reporting and recall bias. Secondly, not all Oto-HN surgeons are members of the CSO-HNS. We designed our survey to be short in order to promote participation; however, this meant that no validated items were used to ask about current practices or beliefs. Furthermore, due to our study design, the actual incidence of occult malignancy in routine specimens could not be measured. We did not ask participants to provide proof of their reported rates of occult malignancy, nor did we ask about whether any participants had experienced litigation as a result of missing an occult malignancy. Therefore, we were unable to comment on the cost of a missed diagnosis compared to the cost of routine specimen analysis. These factors may have impacted our study’s external validity.

## Conclusion

Overall, sending tonsil specimens for HP analysis when no malignancy is suspected is performed across Canada, although this practice is no longer supported by the literature. The majority of the participants in our study were in favour of eliminating this practice in adults and pediatrics. Instead of routinely sending tonsil specimens for evaluation, an alternative approach is to assess patients pre-operatively for factors that increase suspicion of malignancy. This would maintain patient safety while decreasing unnecessary resource use in Canadian healthcare.

## Supplementary Information

**Additional file 1**. Appendix I - Survey Questions for the questions that we asked participants.

## Data Availability

The anonymous survey responses are available from the corresponding author on reasonable request.

## References

[1] Quality-based procedures clinical handbook for paediatric tonsillectomy and adenoidectomy. Ministry of Health and Long-Term Care & Provincial Council for Maternal & Child Health 2016. http://www.health.gov.on.ca/en/pro/programs/ecfa/docs/qbp_tonsil.pdf. Accessed 29 Dec 2020.

[2] Mitchell RB, Archer SM, Ishman SL (2019). Clinical practice guidelines: tonsillectomy in children (update). Otolaryngol Head Neck Surg.

[3] Courville EL, Lew M, Sadow PM (2011). Routine evaluation of adult tonsillectomy specimens: toward establishing a new standard of care. Int J Surg Pathol.

[4] Tran AHL, Chin KL, Horne RSC, et al. Hospital revisits after paediatric tonsillectomy: a cohort study. J Otolaryngol – Head & Neck Surg. 2022;51(1). 10.1186/s40463-021-00552-8 PMC8756632 35022073

[5] Cohan DM, Popat S, Kaplan SE (2009). Oropharyngeal cancer: current understanding and management. Curr Opin Otolaryngol Head Neck Surg.

[6] Saab SS (1998). The cost-effectiveness of routine histologic examination. Am J Clin Pathol.

[7] Damjonov I, Vranic S, Skenderi F (2016). Does everything a surgeon takes out have to be seen by a pathologist? A review of the current pathology practice. Virchows Arch.

[8] Schrock A, Jakob M, Heukamp L (2009). Histology after tonsillectomy?. HNO.

[9] Rokkjaer MS, Klug TE (2014). Malignancy in routine tonsillectomy specimens: a systematic literature review. Eur Arch Otorhinolaryngol.

[10] About Choosing Wisely Canada. Choosing Wisely Canada. https://choosingwiselycanada.org/about/. Accessed 10 March 2021.

[11] McDonough M, Hathi K, Corsten G, et al. Choosing Wisely Canada – pediatric otolaryngology recommendations. J Otolaryngol – Head Neck Surg. 2021;50(61). 10.1186/s40463-021-00533-x PMC8557011 34715936

[12] Burns KE, Duffett M, Kho ME (2008). A guide for the design and conduct of self-administered surveys of clinicians. CMAJ.

[13] Thoma A, Cornacchi SD, Farrokyar F (2011). How to assess a survey in surgery. Can J Surg.

[14] Chow W, Rotenberg BW (2015). Discontinuing routine histopathological analysis after adult tonsillectomy for benign indication. Laryngoscope.

[15] Randall DA, Martin PJ, Thompson LDR (2007). Routine histologic examination is unnecessary for tonsillectomy or adenoidectomy. Laryngoscope.

[16] Three things physicians and patients should question in Otolaryngology— – Head and Neck surgery. Choosing Wisely Canada. www.choosingwiselycanada.org/otolaryngology/. Accessed 10 March 2021.

