# Supplementary material for: Routine evaluation of tonsillectomy specimens: a cross-sectional survey of Canadian Otolaryngology: Head and Neck Surgeons
Source: J Otolaryngol Head Neck Surg. 2022 Apr 6;51:14. doi: 10.1186/s40463-022-00569-7 (PMC8985276; doi:10.1186/s40463-022-00569-7)
Supplement: Supplementary file 1 — Additional file 1. Appendix I - Survey Questions for the questions that we asked participants. [file 40463_2022_569_MOESM1_ESM.docx]

Clinical Practices of Canadian Otolaryngology – Head and Neck Surgeons Surrounding Routine Evaluation of Tonsillectomy Specimens: A Cross-Sectional Survey

**Survey Questions**

**Demographics**

1. Where do you practice Otolaryngology – Head and Neck Surgery most of the time? (participants can select one)
   1. Alberta
   2. British Columbia
   3. Manitoba
   4. New Brunswick
   5. Newfoundland and Labrador
   6. Nova Scotia
   7. Ontario
   8. Prince Edward Island
   9. Quebec
   10. Saskatchewan
   11. Northwest Territories
   12. Nunavut
   13. Yukon
   14. Other: _______________ (open ended)
2. Do you work at an Academic Institution? (participants can select one)
   1. Yes
   2. No
3. What is your age range? (participants can select one)
4. <30
5. 30-39
6. 40-49
7. 50-59
8. 60-69
9. >70
10. Prefer not to answer
11. What is your gender?
    1. Male
    2. Female
    3. Other: _____________ (open-ended)
    4. Prefer not to answer
12. Have you completed fellowship training? (participants can select one)
    1. Yes
    2. No
    3. Not specified
13. How long have you been practicing Otolaryngology – Head and Neck Surgery? (participants can select one)
14. <5 years
15. 5-9 years
16. 10-20 years
17. >20 years

**Clinical Practice**

1. Approximately how many tonsillectomies do you perform annually? (open-ended)
2. Do you perform:
   1. Adult tonsillectomies only
   2. Pediatric tonsillectomies only
   3. Both pediatric and adult tonsillectomies
   4. I do not perform tonsillectomies
3. Do you send tonsil specimens for evaluation when NO malignancy is suspected? (participants can select one)
   1. Yes, for pediatric tonsillectomies only
   2. Yes, for adult tonsillectomies only
   3. Yes, for both adult and pediatric tonsillectomies
   4. No
   5. Not applicable
4. If yes, is this an institutional policy? (participants can select one)
   1. Yes
   2. No
5. If yes, how many specimens to your recollection have returned with occult malignancy in your years of practice? (open-ended)

**Beliefs/Attitudes**

Please rate your agreement with the following statements:

1. In pediatrics, tonsil specimens should be evaluated even when no malignancy is suspected. (participants can choose strongly disagree, disagree, neither agree nor disagree, agree and strongly agree)
2. In adults, tonsil specimens should be evaluated even when no malignancy is suspected. (participants can choose strongly disagree, disagree, neither agree nor disagree, agree and strongly agree)
3. Please write any additional comments you have about this topic. (open-ended)
